# Effect of flipped classroom methodology on the student performance of gastrointestinal and renal physiology entrants and repeaters

**DOI:** 10.1186/s12909-020-02329-5

**Published:** 2020-11-02

**Authors:** Julio C. Sánchez, Diego F. López-Zapata, Óscar A. Pinzón, Andrés M. García, Martha D. Morales, Samuel E. Trujillo

**Affiliations:** Faculty of Health Sciences, Universidad Tecnológica de Pereira, AA 97, La Julita, Pereira, Risaralda, Colombia

**Keywords:** Gastrointestinal and renal physiology, Flipped teaching, Academic stress, Medical students

## Abstract

**Background:**

Physiology is a subject that is considered difficult; it is associated with academic failure and causes high levels of stress and anxiety in students.

**Methods:**

This study compared the effectiveness of a traditional lecture-based methodology with that of a flipped classroom scheme focusing on cooperative ludic learning among gastrointestinal and renal physiology students. Two groups were subjected to these two different methods to teach gastrointestinal and renal physiology content divided into 14 topics. Additionally, two subgroups were identified in each group: entrants and repeaters. There were no differences in age or gender between the subgroups.

**Results:**

Levels of self-perceived stress (measured by the SISCO scale), biological stress (measured by awakening salivary cortisol levels), and anxiety (measured by the Zung scale) were high in all of the students; the cortisol levels increased in the entrants and some of the scores in SISCO scale increased in the repeaters, throughout the study. The self-reported study time was longer in the students subjected to the flipped classroom-based method. The final exam results were better only in the new students facing the flipped methodology, but not in the repeaters, who scored lower on the final evaluation. The quantitative and qualitative assessments completed by the participants regarding the different aspects of the flipped-classroom-based methodology were favorable; however, the participants believed that traditional lectures should be maintained for specific topics.

**Conclusions:**

A methodology based on flipped teaching was an effective strategy to improve academic performance ingastrointestinal and renal physiology, but only in new students.

**Supplementary Information:**

**Supplementary information** accompanies this paper at 10.1186/s12909-020-02329-5.

## Background

Physiology is a subject that belongs to the basic nucleus of knowledge in all health sciences and advancements in key physiological mechanisms continue to occur. This fact poses a challenge to educators to effectively disseminate this complex content to students and contribute to their active engagement in the learning process, which is necessary to promote critical thinking and self-directed learning skills [1]. For these reasons, physiology courses are recognized as difficult and are associated with high levels of withdrawal and failure [2–5]. In our medical school, physiology students also have high rates of failure and repetition, leading to student frustration and loss of motivation and contributing to increased student permanence and desertion [6], as well as generating high stress levels among medical students [7, 8] and resulting in anxiety and depression [9, 10] which can affect academic and social performance [11]. In such a framework it is a challenge to better understand the learning-teaching process in physiology and propose different methodologies, that focus on personalized teaching to improve academic performance [12].

Traditional lectures remain a predominant instructional strategy in physiology education, but this methodology has been reported to be ineffective in many ways because students’ attention diminishes quickly, they frequently feel disengaged, and the lecture rhythm cannot be adjusted to specific cases. Fortunately, options available to approach teaching and learning have evolved with recent advances in technology, allowing teachers and students to find methods that suit their own abilities and time availability; these methods engage students more effectively, [13], provide opportunities to more efficiently use time with teachers [14] and can help teachers to communicate knowledge at the appropriate pace for each student [15]. These features are important particularly in physiologybecause learning in this area requires a transformation in reasoning, involving evolution from more concrete to considerably abstract thinking, which is facilitated by participative methods.

Teaching based on a flipped classroom approach [16] occurs when students perform a significant amount of preparation before class, while the class time, when teachers are present, is reserved for discussion and/or problem solving related to the most important topics [17]. Currently, there is substantial interest in the flipped format in science teaching [18], particularly in fields with high levels of difficulty in learning [19]. Moreover, there is growing interest in the implementation of cooperative learning, which occurs when students work together in groups to reach their learning goals through discussion and peer feedback, usually under teacher supervision [19]; students involved in cooperative learning show greater effort to achieve than students who learn on their own [20]. Cooperative learning could be incorporated into the flipped classroom methodology by encouraging students to form teams and by assigning homework to complete in groups.

Another component that is important to consider is the incorporation of methodologies involving ludic engagement [21], since these methodologies can help students to learn in a less hostile environment. Playing and learning in a different and fun context can help students to understand concepts and could help to create bonds between classmates, which reinforce cooperative learning.

The purpose of this study was to compare the effectiveness of a traditional lecture-based methodology versus a flipped classroom scheme focused on cooperative ludic learning in entrants and repeaters. The subject assessed was gastrointestinal and renal physiology, which was delivered to second-year medical students at the Universidad Tecnológica de Pereira during 2018 and 2019.

## Methods

### Subjects

The participants were all second-year medical students who were enrolled in the Medical Physiology course in 2018–2019 and agreed to participate in the study. There were no refusals to do so. Two separate successive nonrandomized groups of students were considered, according to the distribution normally made by the university, which cannot be altered for institutional reasons. The first group was assigned to a traditional lecture-based learning methodology (TG) and was studied first. The second group was assigned to a flipped teaching methodology (FCG), focusing on cooperative learning and alternative ludic strategies and was studied in the subsequent academic period. This design avoided the influence of components of the flipped classroom methodology applied to FCG on TG. The sample was composed of 75 students; 46 were exposed to the traditional strategy (TG, age: 23 ± 3 years old, 52% female) and 29 were exposed to the flipped classroom methodology (FCG, age: 21 ± 3 years old, 55% female). There were 15 students in the TG (TG1, age: 23 ± 3 years old, 52% female) and 10 students in the FCG (FCG1, age: 22 ± 3 years old, 52% female) who were exposed to the course for the first time, defined as entrants. All of the other students (TG2: 31, age: 23 ± 3 years old, 51% female. FCG2: 19, age: 21 ± 3 years old, 52% female) had been enrolled at least once before in the traditional course and they were defined as repeaters (with an average of 3 ± 1 times). There were no differences between groups or subgroups in gender or age distribution. However, the group of entrants had significantly better grades in previous courses (3.75 ± 0.25) in comparison with the group of repeaters (3.26 ± 0.28); there were no differences between grades when comparing TG1 (3.71 ± 0.31) and FCG1 (3.79 ± 0.22) or TG2 (3.27 ± 0.29) and FCG2 (3.25 ± 0.28). All of the participants signed an informed consent form and the study was approved by the Ethics Committee of the Universidad Tecnológica de Pereira.

### Traditional methodology

The TG was subjected to a traditional lecture-based methodology. Fourteen two-and-a-half-hour-long lectures were developed to deliver the topics of gastrointestinal and renal physiology (Table 1). At the end of each lecture there was a 30-min session for questions and answers about the topic of the day. A bibliography was recommended to all of the students. Since the TG course was conducted first, this group had no access to any of the tools designed to the FCG, which were implemented during a different period of time.

**Table 1:** Previous knowledge and English proficiency test scores in all groups. Data are expressed in medians and interquartile ranges of percentages. FTS: first time students. RS: repeating students. TG: Traditional Group. FCG: Flipped Classroom Group. PK: Previous knowledge. ERP: English reading proficiency

Test	FTS		RS	
	TG	FCG	TG	FCG
PK	37 (31.8–47)	32 (27–42)	32 (25–40)	35 (30–40)
ERP	61 (39–73)	56 (33–70)	50 (43–71)	40 (26–59)

### Flipped classroom methodology

The FCG was subjected to a flipped classroom methodology focused on cooperative learning. The content was divided into the same 14 topics as in the traditional methodology, but there were no lectures. For each topic, a 20- to 30- slide presentation was designed by the teachers, and a short review article about a relevant topic was selected from a high-quality journal; these materials were delivered to the students before the course began, and each topic was assigned to a specific date on the schedule. The students were instructed to review the materials and the additional recommended readings from the bibliography and to discuss them with others before the assigned date. During class time (3 h for each session), the students were encouraged to form teams to discuss the corresponding topic and ask all of their questions to the teachers, who guided the discussions in each group. At the end of each session, the students were assigned an activity to assess their knowledge and facilitate complementary learning about the specific topic; the assignments involved the students playing games (crosswords, word searches, quiz contests, question and answer games) that allowed all of them to participate in a ludic way in the process. Every day, the students were assigned a 10-question quiz about the topic developed on the previous day. Additionally, a challenge about the topic of the day was posed to the students to be solved voluntarily; these challenges involved interpretation of the literature, information searches about a related subject, developments of a historical perspective, literary interpretations of physiological concepts or completion of specific exercises that had to be answered in a limited amount of time. At all times, a virtual platform was available for the students to communicate with their teachers and classmates; they could use the platform to formulate questions, write reflections or express opinions about the different topics, which could be commented on or answered by everyone, particularly the teachers. This platform was freely accessible to all the participants, but restricted to them only and it worked based on a very friendly environment, similar to other social networking sites, which are commonly used by young adults.

### Assessment of previous knowledge

An examination was designed with questions that assessed biomedical subjects from courses that were academic prerequisites for the Medical Physiology course and that had been passed by all of the students before entering the course; these subjects included biology, biochemistry, molecular biology, histology and anatomy. The examination was delivered to both groups before starting the study. The scores were reported as percentages of the total possible score.

### Assessment of previous English reading skills

Given that all of the scientific literature was delivered in English and all of the students spoke Spanish as their native language, an examination to evaluate English reading skills was designed and administered to all of the students before the study. The scores were reported as percentages of the total possible score.

### Stress and anxiety assessment

To evaluate stress levels, two strategies were employed. The first was the measurement of awakening salivary cortisol as an effective biomarker for stress [22, 23]; salivary cortisol levels are correlated with serum cortisol levels, but their normal range can vary due to ethnicity and individual backgrounds [24]. A normal interval for teenagers was reported recently for cortisol awakening response of 5–23 μg/dL; nevertheless, it is only a mean for understanding stress responses and does not address specific medical issues [25]. Therefore, three saliva samples (1 mL each) were obtained from each student: the first sample was collected at baseline, and the other two were collected in the middle and at the end of the study to evaluate changes in this biological stress marker during the study. The samples were stored at − 20 °C until the cortisol concentrations were determined. Salivary cortisol levels were measured using a commercially available ELISA kit (Cortisol Saliva KAPDB290 DIA Source, Louvain-la-Neuve Belgium).

The second strategy was the administration of the SISCO scale [26] to measure self-perceived academic stress; the scale was administered 3 times on the same days on which the students provided the saliva samples. The SISCO scale consists of 6 dimensions, including intensity of academic stress (A), environmentally stressful stimulus (B), physical reactions (C), psychological reactions (D), behavioral reactions (E) and coping strategies (F); all of the dimensions are scored between 1 and 5. This scale has been validated in Spanish and has been employed in Colombian students [27]. All of the dimensions are scored between 1 to 5, with the highest score being a more severe form of stress, except for coping strategies for which a greater score represents a better adaptative response to stress [28, 29].

Anxiety levels were assessed employing the Zung anxiety scale [30], which has been validated in Spanish and applied to Colombian university students [31]. This test was also administered 3 times, on the same days on which the students provided the saliva samples and completed the SISCO scale. The total raw scores range from 20 to 80, and it classifies the symptoms using the score obtained: 20–44 normal, 45–59 mild, 60–74 moderate and 75–80 severe [32].

### Self-reported study time

Students were instructed to report the amount of time that they invested in academic activities every day, and an the average number of minutes that each participant spent studying was calculated.

### Assessment of acquired knowledge

After the 14 topics were covered, all of the students completed an examination that covered all aspects of renal and gastrointestinal physiology, which was designed by the teachers who were involved in the process. This exam was applied to Group 1 and Group 2 in different times, at the end of the course, but it had the same characteristics and both groups had the same amount of time to prepare for the evaluation. The exam contained 40, 4-option multiple choice questions. The test maintained a balance between questions that assessed memory of facts and questions that assessed application of knowledge. The final scores were between 0.0 and 5.0 according to the scale employed in the academic institution to which the students belonged.

### Students’ opinions

An instrument was designed to collect the participants’ opinions about the main aspects included in the flipped classroom methodology (FCG group), which was completed by the students following the final examination. The first part asked the participants to quantitatively evaluate 17 aspects of the methodology by rating them between 0 and 5, with 5 being the most positive evaluation (Table 4). Additionally, the participants were asked to answer the two following open-ended questions.

1. What are your opinions (positive and negative) about all of the aspects of the flipped classroom methodology?
2. Do you have any suggestions about it?

### Statistical analysis

All of the variables were evaluated for normal distribution with the Shapiro-Wilk test, and the only variable that had a normal distribution was the quantitative student assessment of the flipped classroom methodology. The Mann-Whitney test was conducted for each variable to establish the statistical significance of the differences between the traditional and flipped classroom model. The Kruskal-Wallis test and Dunn’s multicomparison test were also used to compare all of the variables together. For repeated measures analysis between the follow-up measurements in the same group (TG or FCG) Friedman’s test and Dunn’s multicomparison test were used. All of the analyses were performed using GraphPad Prism 8 (GraphPad Software Inc., La Jolla, CA, USA). *p* < 0.01 was considered significant for all of the tests.

### Qualitative analysis

All of the qualitative information provided by the students was analyzed employing grounded theory [33, 34]. All of the information was processed by extracting the emerging themes and identifying categories based on the information supplied. This analysis was performed in Spanish first and then the condensed information was translated into English.

## Results

Given the characteristics of each group, the analysis was conducted by comparing the data obtained from entrants and repeaters in both groups (TG1 vs FCG1 and TG2 vs FCG2).

### TG1 vs FCG1

There were no significant differences in the previous knowledge test and English reading test scores (Table 1). The scores on the baseline assessment of previous knowledge that is considered a prerequisite for any physiology course were low in both groups (less than 40%), and the results of the English reading test were poor (between 40 and 60%), considering the importance of English reading to accessing knowledge in physiology.

The levels of salivary cortisol, anxiety and self-perceived academic stress were high in both groups, as shown in Table 2. The salivary cortisol concentrations showed no differences between groups in any of the quantifications, but the second measurement was significantly higher than the first one in both groups, without additional increases.

**Table 2:** Stress and anxiety assessment in TG1 (Traditional Group 1) and FCG1 (Flipped Classroom Group 1). IQR: Interquartile range. SISCO: Inventory of Academic Stress. Data are expressed in medians and interquartile ranges. * denotes significant differences in comparison to the first measurement in the same group (p < 0.01). A: Intensity of academic stress. B: Environment stressful stimulus. C: Physical reactions. D: Psychological reactions. E: Behavioral reactions. F: Coping strategies

		TG1 (*n* = 15)			FCG1 (*n* = 10)		
Parameter / Follow-ups		1	2	3	1	2	3
Cortisol (μg/dL)		31 (17–50)	57 (41–89)*	41 (24–80)	29 (21–80)	48 (26–85)*	48 (24–104)
Zung scale		40 (30–44)	37 (30–42)	37 (32–44)	37 (33–44)	43 (32–50)	43 (32–48)
SISCO	A	4 (3–4.25)	4 (3–4)	4 (4–5)	4 (3–4)	4 (3–4)	4 (3–5)
	B	2.7 (2.4–3.1)	2.7 (1.9–3.3)	2.6 (1.9–3.2)	3.2 (2.5–3.6)	3.5 (2.7–4.3)	3.5 (2.7–3.7)
	C	2.6 (1.5–2.9)	2.3 (1.7–3.2)	2.3 (1.8–3)	2.7 (1.8–3.3)	3.2 (1.8–3.6)	3.2 (2.3–3.5)
	D	2.7 (2.1–3.6)	2.7 (2.1–3.1)	2.8 (2.5–3.1)	3.2 (2.4–3)	3 (2.6–4.4)	3 (2.6–3.6)
	E	2.7 (2.2–3.0)	2.6 (2.0–3.1)	3 (2.4–3.3)	2.7 (2.3–3)	2.7 (2.5–3.5)	3 (2.3–3.3)
	F	2.8 (2.6–3.7)	3.3 (2.6–3.7)	3 (2.5–3.8)	2.8 (2.5–3.1)	2.8 (2.3–3.6)	2.7 (2.6–3.3)

There were no significant differences in anxiety scale scores or self-perceived stress scores (in any of the dimensions) between TG1 and FCG1, and in addition, there were no differences when comparing the three successive measurements that were obtained.

**Fig. 1:**
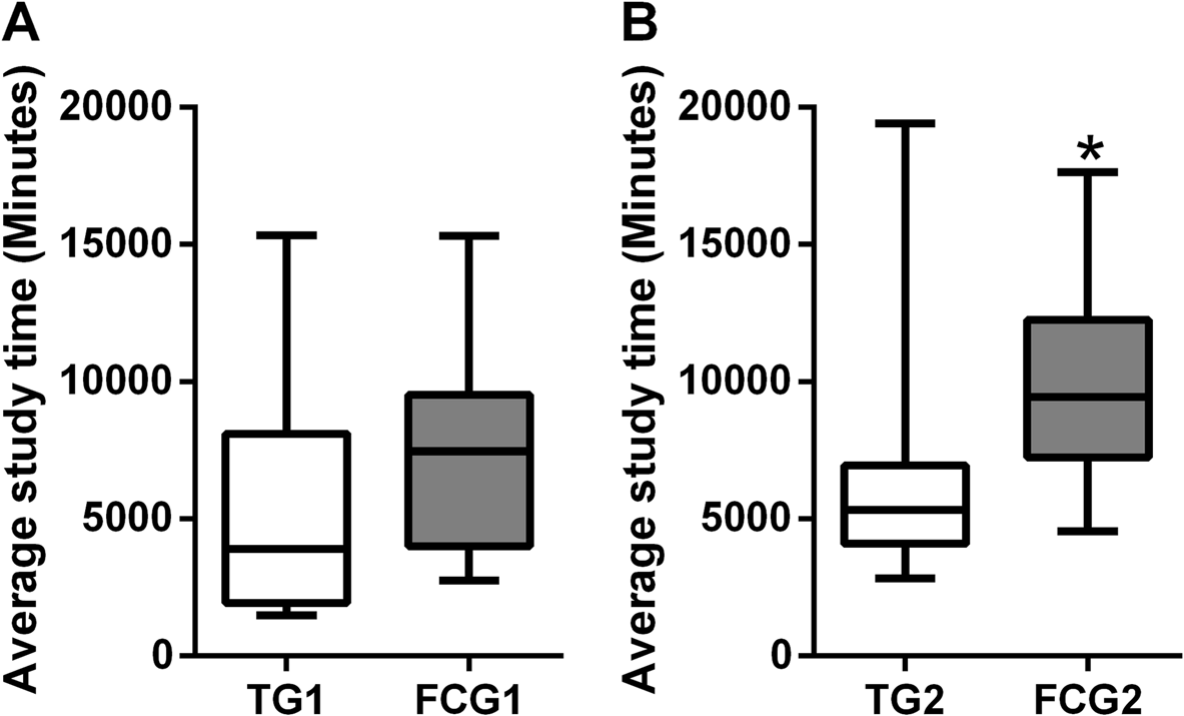
Average daily study time. Panel **a** shows the TG1 vs FCG1 comparison. Panel **b** shows the TG2 vs FCG2 comparison. Data are expressed in medians and interquartile ranges. *p < 0.05. TG: Traditional Group. FCG: Flipped Classroom Group

Self-reported study time was not significantly different between groups (TG1: median 3909, IQR 1930–8096 vs. FCG1: median 7452, IQR 3996–9520). A high dispersion of the data was observed, particularly in TG1 (Fig. 1a).

**Fig. 2:**
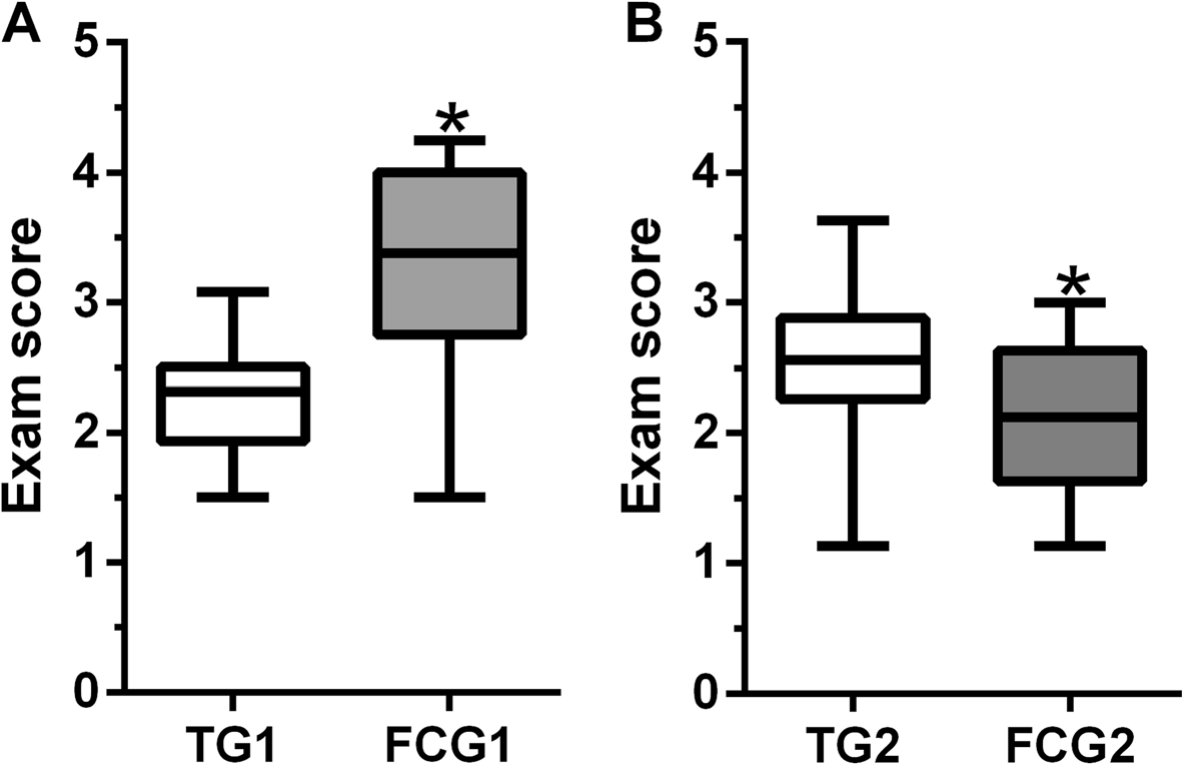
Results of the final test. Panel **a** shows the TGC1 vs FGC1 comparison. Panel **b** shows the TG2 vs FCG2 comparison. Data are expressed in medians and interquartile ranges. *p < 0.01. TG: Traditional Group. FCG: Flipped Classroom Group

The final examination scores were significantly higher in FCG1 (median: 3.38, IQR 2.75–4.0) than in TG1 (median: 2.31, IQR 1.93–2.5) (Fig. 2a).

### TG2 vs FCG2

There were no significant differences in the previous knowledge test and English reading test scores (Table 1). As observed in TG1 and FCG1, both scores on the baseline assessment were deficient.

There were no significant differences in salivary cortisol concentrations and the Zung scale scores between TG2 and FCG2, although both parameters were high at the beginning of the study. It is worth noting that the initial levels of salivary cortisol were significantly higher in TG2 and FC2 in comparison with TG1 and FCG1, although after the second measurement, there were no differences between groups. The self-perceived stress scores were also high in general and the first measurements were significantly lower than the other two measurements in TG2 in all of the dimensions, except for the A dimension, which, was nevertheless scored with very high punctuation in both groups (Table 3). In contrast, in FCG the dimensions in which the score was higher in the second measurement were B and E; in all of the others the scores were high since the beginning.

**Table 3:** Stress and anxiety assessment in global TCG2 (Traditional Group 2) and FCG2 (Flipped Classroom Group 2. IQR: Interquartile range. SISCO: Inventory of Academic Stress. Data are expressed in medians and interquartile ranges. * denotes significant differences in comparison to the first measurement in the same group (p < 0.01). A: Intensity of academic stress. B: Environment stressful stimulus. C: Physical reactions. D: Psychological reactions. E: Behavioral reactions. F: Coping strategies

		TG2 (*n* = 31)			FCG2 (*n* = 19)		
Parameter / Follow-ups		1	2	3	1	2	3
Cortisol (μg/dL)		66 (44–86)	72 (50–87)	52 (44–70)	97 (59–125)	64 (39–99)	77 (57–123)
Zung scale		41 (38–48)	43 (38–50)	48 (42–57)	40 (36–48)	47 (43–54)	50 (44–56)
SISCO	A	4 (3–4.2)	4 (3–4)	4 (4–5)	4 (3–4)	4 (3–4)	4 (3–5)
	B	2.6 (2.2–3.1)	3.2 (3.1–4)*	3.2 (3–4)	3.1 (2.7–3.3)	3.7 (3.3–4.1)*	3.6 (3.3–4.1)
	C	2.3 (1.8–2.8)	3.1 (3–3.3)*	3.5 (2.8–4)	3.1 (2.5–3.3)	3.3 (3–3.8)	3.3 (3–3.8)
	D	2.2 (2–2.8)	3.4 (2.8–3.8)*	3.8 (3.2–4.2)	3.2 (2.8–3.4)	3.4 (3–3.8)	3.6 (3.2–4.2)
	E	1.7 (1–2)	2.7 (2.5–3.2)*	2.7 (2.2–3.2)	2.7 (2.5–3)	3.5 (2.9–3.7)*	3 (2.7–3.7)
	F	2.3 (1.8–2.8)	3.1 (3.1–3.5)*	3.3 (3–3.6)	2.8 (2.5–3.3)	3 (2.8–3.5)	3.1 (2.6–3.6)

Self-reported study time was significantly different between groups (Fig. 1b), being longer in FCG2 (median: 9428, IQR 7238–12,260) in comparison to TG2 (median: 5315, IQR 4092–6959).

The final examination scores were significantly lower in FCG2 (median: 2.12, IQR: 1.63–2.63) than in TG2 (median: 2.56, IQR: 2.26–2.88) (Fig. 2b).

### Student opinions

#### Quantitative evaluation

Table 4 shows the results for the seventeen aspects the participants evaluated. The data were the assessments of all of the participants exposed to the flipped classroom methodology, since there were no significant differences between new students and repeaters. The only aspect that received a score less than 3.0 was classroom ventilation. All of the other items were evaluated with scores higher than 3.4 and 7items had scores higher than 4.0.

**Table 4:** Quantitative student assessment of the flipped classroom methodology, rated between 0 and 5, with 5 being the most positive evaluation. Data are expressed in means and standard deviations

Issue	Score, *n* = 29
Content	4.3 ± 0.8
Organization	4.6 ± 0.7
Presentations quality	3.7 ± 1.1
Blog	3.9 ± 0.8
Chat	3.6 ± 1.5
Forum	3.57 ± 1.2
Group tutorials	3.5 ± 1.4
Individual tutorials	4.1 ± 1.2
Daily activities	3.4 ± 1.2
Short exams	3.9 ± 0.9
Final exam	4.0 ± 1.1
Classroom illumination	4.4 ± 0.5
Classroom ventilation	2.5 ± 0.9
Classroom cleanness	4.5 ± 0.6
Audiovisual support	3.7 ± 0.9
Complementary material	3.8 ± 1.1
Bibliography	4.2 ± 1.1

#### Qualitative evaluation

The results of the open qualitative assessment performed by the students subjected to the flipped methodology were grouped according to the following emergent themes: methodology, evaluation system, activities and challenges. Three dimensions were identified within each theme: negative opinions, positive opinions and recommendations.

##### Methodology

The students had positive views of the flipped classroom methodology because they thought that it compelled them to be active subjects by reading and informing themselves much more about the topics; in addition, they thought that the daily evaluations facilitated continuous study. One student said: “The opportunity to develop the subjects daily allows us to build better knowledge and develop more solid concepts”. The students confirmed that the flipped classroom methodology trained them to better manage their study time, and that, through the process, they acquired a higher level of understanding of the subject; additionally, they described the methodology as “demanding, creative, participative and motivating”. In this respect, one of the students expressed that “the methodology was very effective, motivating the student and contributing to her/his independence”. This observation reinforces the personalized aspect of the methodology, which stimulates students to become active subjects who are responsible for their own learning. Another positive opinion concerned the papers and bibliographic material employed, which were evaluated as “current, interesting, useful and appropriate”.

The negative opinions referred mainly to the inadequate allocation of time to some of the topics. One of the students mentioned that “the available time for some of the more extensive topics did not allow us to work on them properly”. Additionally, some students expressed that it was impossible to include all of the topics in the period of time assigned and recommended not excluding traditional lectures from the methodology, because they considered these classes to be necessary, especially for some specific topics. The main suggestion was to offer a class focusing on correcting mistakes, clarifying doubts and avoiding incorrect interpretations of formal texts. These observations indicated that the majority of the students (80%) recommended the inclusion of traditional classes guided by the teacher.

##### Evaluation system

The favorable opinions expressed in the evaluations noted that the daily short exams compelled the students to study continuously and review the topics before the sessions with the teacher. One of the students explained that “the exams make it necessary to study all the themes daily”, and another expressed that “daily evaluations obligate students to be studying all the time to have all the topics fresh and current”.

Regarding the negative opinions, the students emphasized the short time available to complete the exams, the sense of competition that emerged among students to obtain better scores which generated a negative environment and teacher subjectivity in some evaluations.

##### Activities and challenges

According to the students, the new activities contributed to a different and exciting environment for learning physiology and promoted teamwork through the stimulation of collaborative learning, which broke the routine. The students commented that “all the activities and challenges were nice and interactive”.

A negative aspect concerned the short time for participating in the activities and challenges, which led to them not solving some of the challenges. In addition, some students said that the challenges were too numerous and too difficult and required a long time to complete, preventing them from properly preparing for the topics on the next day.

## Discussion

This study assessed the effectiveness of a traditional lecture-based methodology with that of a flipped classroom scheme focusing on cooperative ludic learning in medical students enrolled in a gastrointestinal and renal physiology course, divided into two groups: entrants and repeaters. The flipped classroom methodology improved the final outcome only in the entrants. These two groups showed significant differences, not only because of their relationship with the physiology course but also because the new students exhibited better previous academic performance and lower initial levels of salivary cortisol. It is worth noting that, in our medical school, the rate of repetition in physiology courses is a great problem; in the last five years, in each academic semester, 60 ± 9% of the students registered in the Medical Physiology course were repeaters, and they exhibited consistently poor academic performance (data from official academic records).

All of the students included in this study showed low scores in prerequisite knowledge and English reading, indicating that they had insufficient academic tools to engage in a physiology course, which requires solid concepts in biology, biochemistry, molecular biology, histology and anatomy [35], plus a good level of English reading proficiency to achieve the goals of the teaching-learning process in physiology. This fact could be one of the causes of the difficulty that students perceive with physiology [5], which generates a high level of stress even before starting the course. This finding must motivate us to reinforce strategies to optimize learning in previous courses and to use methods to ensure that students have the skills that they need to face physiology programs.

The levels of stress and anxiety among Colombian medical students are high, and being enrolled in basic subjects is a risk factor for higher levels of stress and the development of emotional complications [10, 36]. In this study, the initial levels of anxiety, self-perceived academic stress and biological stress were consistently high among all of the participating students. These findings showed that physiology courses could be stressful and that these factors must be considered part of the teaching-learning process, which will require the inclusion of different strategies to give students a way to cope with stress and anxiety, which can generate important psychological and physical loads that can affect academic performance [37] and, over all, have a significant effect on student wellbeing [38, 39].

The students in FCG1 performed significantly better on the final exam than the students in TG1, consistent with the results of a number of studies that evaluated the effects of participative strategies on performance in different health sciences fields [17, 40–42], particularly in physiology [15, 43]. TG1 and FCG1 students showed high levels of stress and anxiety, as did all of the other students, but these students were new to the course, which distinguished them from the others. In these students, the only parameter that changed during the study was the level of salivary cortisol, which increased significantly in the second measurement, without a further increase, which could reflect the biological stress secondary to facing a completely different methodology, although the flipped classroom strategy had a positive effect on their final performance, possibly because the course was their first contact with these specific topics, and the participative method gave them new tools to better face the challenge of learning specific concepts. Additionally, the availability of a virtual platform that allowed them to communicate and interact with teachers and peers permanently helped them to learn more effectively, as they reported in the qualitative evaluation.

In contrast, the students in TG2 performed significantly better on the final exam than the students in FCG2, although they reported dedicating less time to studying. Their worse performance when subjected to the flipped classroom methodology could be a consequence of exposing students with poor previous knowledge and academic outcomes to a new method that requires many tools to perform it efficiently. Therefore, the FCG2 students had to spend more time trying to learn the material by themselves, which required some autonomy and not only reading class notes and following a rigid guide, which was the format with which they were familiar from other courses. Students prefer to adapt old strategies to a new method because it is familiar and because there is some resistance to change [44], since adapting to new methods requires more time and generates more stress. Another possibility to consider is that the students in FCG2 were too confident about their previous knowledge because they were repeaters and for this reason, they did not study the materials or participate actively in the flipped classroom strategy. Due to familiarity, the TG1 students were acquainted with performing with known conditions and content. However, the general performance was low in both TG2 and FCG2, consistent with the academic history of the subjects included in these groups. It is worth noting that, although in all of the participants the levels of salivary cortisol were high, the initial levels were significantly higher in repeaters, which is a factor that must be considered a contributor to their poor performance. Furthermore, second measurement of subjective stress levels increased in some dimensions in repeaters, perhaps due to expectations about the proximity of the final examination, which could significantly affect their final grades. These findings could be explained by the repeaters, in contrast with the new students, having higher expectation levels because they had had previous contact with the content and perceived the process to be very difficult.

In general, all of the students, without differences between entrants and repeaters, evaluated participating in the flipped classroom methodology to be a positive experience, as evidenced by the qualitative and quantitative assessments, in agreement with other studies [19, 42]. The only aspect that was consistently poorly evaluated was the ventilation of the room in which the encounters with the teacher occurred, which was a location problem, that underscored an important issue to resolve. Another issue to be noted was student complaints about insufficient time to spend in independent work, which should be considered when designing academic schedules.

The students emphasized the positive experience when enjoyable activities were included, consistent with other reports [40]. All of the other aspects were considered positive, but the majority expressed the need to maintain traditional lectures, in which the teacher is present and explains the topic to the students, at least for some specific themes. This outcome indicates that, for the heterogeneous groups in our institutions, it is necessary to design courses that employ different methodologies and strategies and that combine participative and traditional methods.

Continuous evaluation is a very positive aspect of the new approach, since the students identified this strategy as a way to compel students to dedicate more time to studying the topics. These observations have been supported by a number of authors, who have argued that formative evaluations allows for the detection of progress and difficulties in the teaching-learning process, enabling teachers and students to progressively adjust the process [45]. Additionally, creating new ways to talk about topics, employing ludic strategies and using challenges and contests to prompt students to work by themselves and to interact with their peers were positive aspects according to the majority of the participants; the main problem identified by the participants was the amount of time because these types of activities require more dedication, and thus, methods based on these strategies require more time, which can be a problem in some curricular structures.

The limitations of this study include that the reliability of some answers depends on the backgrounds, the grades of training and the cognitive development of the students, which we did not measure. Additionally, the sample was small and not randomized, and the groups were not simultaneous because the students enrolled in the courses could not be controlled or changed in any way; thus, the results cannot be extrapolated to the general student population. In this study, we only evaluated gastrointestinal and renal physiology modules, corresponding to the physiology course’s final part; at the time, many students could have already been tired and discouraged because of poor previous academic performance, which this study did not assess.

## Conclusions

A methodology based on flipped teaching was an effective strategy to improve academic performance in new students of gastrointestinal and renal physiology, but the same method was unsuccessful in repeaters. In all of the physiology students, stress and anxiety levels were consistently high, indicating the need to design strategies to help students cope with stress and anxiety. This study contributes to discussions of the necessity for innovation in the methods employed in medical education to optimize the teaching-learning process [46, 47].

## Supplementary Information

**Additional file 1: Table Supplementary.** Theme distribution in the gastrointestinal and renal physiology course.

## Data Availability

All of the data generated or analyzed during this study are included in this published article and any additional information is available from the corresponding author on reasonable request.

## References

[1] Haramati A (2000). Teaching physiology: filling a bucket or lighting a fire. Physiologist..

[2] Harris DE, Hannum L, Gupta S (2004). Contributing factors to student success in anatomy & physiology: lower outside workload & better preparation. Am Biol Teach.

[3] Slominski T, Grindberg S, Momsen J (2019). Physiology is hard: a replication study of students' perceived learning difficulties. Adv Physiol Educ.

[4] Sturges D, Maurer TW, Allen D, Gatch DB, Shankar P (2016). Academic performance in human anatomy and physiology classes: a 2-yr study of academic motivation and grade expectation. Adv Physiol Educ.

[5] Michael J (2007). What makes physiology hard for students to learn? Results of a faculty survey. Adv Physiol Educ.

[6] Carvajal P, Aa T, Mejía J (2006). Deserción estudiantil, Facultad de Ciencias de la Salud 2000–2004. Análisis de correspondencias múltiples. Revista Médica de Risaralda..

[7] Sohail N (2013). Stress and academic performance among medical students. J Coll Physicians Surg Pak.

[8] Singh R, Goyal M, Tiwari S, Ghildiyal A, Nattu SM, Das S (2012). Effect of examination stress on mood, performance and cortisol levels in medical students. Indian J Physiol Pharmacol.

[9] Cardona D, Tabima D, Mejía R (2005). Incidencia y prevalencia de depresión en estudiantes de Ciencias de la Salud de la UTP, 2003. Revista Médica de Risaralda.

[10] Herrera AC, Gómez R (2001). Niveles de ansiedad en estudiantes de Ciencias Básicas de la Facultad de Medicina UTP. Revista Médica de Risaralda..

[11] Voltmer E, Kieschke U, Schwappach DL, Wirsching M, Spahn C (2008). Psychosocial health risk factors and resources of medical students and physicians: a cross-sectional study. BMC Med Educ.

[12] Goldberg HR, Haase E, Shoukas A, Schramm L (2006). Redefining classroom instruction. Adv Physiol Educ.

[13] Freeman S, Eddy SL, McDonough M, Smith MK, Okoroafor N, Jordt H (2014). Active learning increases student performance in science, engineering, and mathematics. Proc Natl Acad Sci U S A.

[14] DeRuisseau LR (2016). The flipped classroom allows for more class time devoted to critical thinking. Adv Physiol Educ.

[15] Montrezor LH (2016). Performance in physiology evaluation: possible improvement by active learning strategies. Adv Physiol Educ.

[16] DeLozier SJ, Rhodes MG (2017). Flipped classrooms: a review of key ideas and recommendations for practice. Educ Psychol Rev.

[17] Pierce R, Fox J (2012). Vodcasts and active-learning exercises in a "flipped classroom" model of a renal pharmacotherapy module. Am J Pharm Educ.

[18] Roehl A, Reddy SL, Shannon GJ (2013). The flipped classroom: an opportunity to engage millennial students through active learning strategies. J Fam Consumer Sci.

[19] Foldnes N (2016). The flipped classroom and cooperative learning: evidence from a randomised experiment. Act Learn High Educ.

[20] Roseth CJ, Johnson DW, Johnson RT (2008). Promoting early adolescents' achievement and peer relationships: the effects of cooperative, competitive, and individualistic goal structures. Psychol Bull.

[21] Selander S (2008). Designs for learning and ludic engagement. Digital Creativity.

[22] Hellhammer DH, Wust S, Kudielka BM (2009). Salivary cortisol as a biomarker in stress research. Psychoneuroendocrinology..

[23] Clow A, Thorn L, Evans P, Hucklebridge F (2004). The awakening cortisol response: methodological issues and significance. Stress.

[24] Dorn LD, Lucke JF, Loucks TL, Berga SL (2007). Salivary cortisol reflects serum cortisol: analysis of circadian profiles. Ann Clin Biochem.

[25] Šupe-Domić D, Milas G, Stanišić L, Drmić Hofman I, Martinović KI (2018). Reference intervals for six salivary cortisol measures based on the Croatian late adolescence stress study (CLASS). Biochemia Medica.

[26] Barraza A. Propiedades psicométricas del Inventario SISCO del estrés académico. Psicología Educativa. 2007;9(13).

[27] Malo DA, Cuadrado E, Florián R, Sánchez D (2011). Análisis psicométrico del inventario SISCO de estrés académico en adultos jóvenes de la Universidad de Sinú In: Barraza A, Jaik A, editors. Estrés, burnout y bienestar subjetivo. Investigaciones sobre la salud mental de los agentes educativos.

[28] Barraza MA (2008). El estrés académico en alumnos de maestría y sus variables moduladoras: un diseño de diferencia de grupos. Avances en psicología latinoamericana.

[29] Macias AB (2007). El Inventario SISCO del estrés académico. Investigación Educativa Duranguense.

[30] Zung WW (1971). A rating instrument for anxiety disorders. Psychosomatics.

[31] De La Ossa S, Martinez Y, Herazo E, Campo A (2009). Estudio de la consistencia interna y estructura factorial de tres versiones de la escala de Zung para ansiedad. Colombia Médica.

[32] Zung WW. The measurement of affects: depression and anxiety. Psychological measurements in psychopharmacology: Karger Publishers; 1974. p. 170–88. 10.1159/000395075 4153516

[33] Glaser BG, Strauss AL (1999). Discovery of grounded theory: strategies for qualitative research.

[34] De la Cuesta Benjumea C (2006). La Teoría Fundamentada como herramienta de análisis. Cultura de los Cuidados.

[35] Rovick AA, Michael JA, Modell HI, Bruce DS, Horwitz B, Adamson T (1999). How accurate are our assumptions about our students' background knowledge?. Am J Phys.

[36] Lemos M, Henao-Pérez M, López-Medina DC (2018). Stress and Mental Health in Medical Students: Relation with Coping and Extracurricular Activities. Archivos de Medicina.

[37] Sandi C, Pinelo-Nava MT (2007). Stress and memory: behavioral effects and neurobiological mechanisms. Neural Plasticity.

[38] Abdulghani HM, AlKanhal AA, Mahmoud ES, Ponnamperuma GG, Alfaris EA (2011). Stress and its effects on medical students: a cross-sectional study at a college of medicine in Saudi Arabia. J Health Popul Nutr.

[39] Caserta MT, O'Connor TG, Wyman PA, Wang H, Moynihan J, Cross W, et al. The associations between psychosocial stress and the frequency of illness, and innate and adaptive immune function in children. Brain Behav Immun. 2008;22(6):933-40. 10.1016/j.bbi.2008.01.007. 10.1016/j.bbi.2008.01.007 PMC2516370 18308510

[40] Baid H, Lambert N (2010). Enjoyable learning: the role of humour, games, and fun activities in nursing and midwifery education. Nurse Educ Today.

[41] Ahmad FA, Karimi AA, Alboloushi NA, Al-Omari QD, AlSairafi FJ, Qudeimat MA (2017). Stress Level of Dental and Medical Students: Comparison of Effects of a Subject-Based Curriculum versus a Case-Based Integrated Curriculum. J Dent Educ.

[42] Herrero JI, Quiroga J (2020). Flipped classroom improves results in pathophysiology learning: results of a nonrandomized controlled study. Adv Physiol Educ.

[43] Abraham R, Ramnarayan K, Kamath A (2008). Validating the effectiveness of clinically oriented physiology teaching (COPT) in undergraduate physiology curriculum. BMC Med Educ.

[44] Olivares Olivares SL, López Cabrera MV, Valdez-García JE. Aprendizaje basado en retos: una experiencia deinnovación para enfrentar problemas de salud pública. Educación Médica. 2018:1–8. 10.1016/j.edumed.2017.10.001.

[45] Perez Michel EJ, Jose C (2017). La evaluación formativa en el proceso enseñanza aprendizaje. Edumecentro.

[46] Bohórquez F, Gutiérrez EF (2004). Modelos pedagógicos y cambios curriculares en medicina: una mirada crítica. Revista Facultad de Ciencias de la Salud Universidad del Cauca.

[47] Daza J. Renovación curricular en programas de ciencias de la salud y su impacto en las prácticas pedagógicas de los profesores Revista de Ciencias de la Salud. 2010;8(1):69–83.

