# Supplementary material for: Effect of flipped classroom methodology on the student performance of gastrointestinal and renal physiology entrants and repeaters
Source: BMC Med Educ. 2020 Nov 2;20:401. doi: 10.1186/s12909-020-02329-5 (PMC7607871; doi:10.1186/s12909-020-02329-5)
Supplement: Supplementary file 1 — Additional file 1: Table Supplementary. Theme distribution in the gastrointestinal and renal physiology course. [file 12909_2020_2329_MOESM1_ESM.docx]

| Topics |
| --- |
| 1. Digestive system organization and control. |
| 2. Mouth and esophagus. |
| 3. Stomach. |
| 4. Exocrine pancreas. |
| 5. Liver, gallbladder and bile duct. |
| 6. Intestine. |
| 7. Corporal fluids and hydric balance. |
| 8. Basic renal processes. |
| 9. Renal perfusion and glomerular filtration. |
| 10. Proximal tubule. |
| 11. Henle loop and distal nephron. |
| 12. Dilution and concentration of urine. |
| 13. Renal regulation of acid-base equilibrium. |
| 14. Micturition, lower urinary tract and renal regulation of homeostasis. |

**Table - Supplementary.** Theme distribution of the gastrointestinal and renal physiology physiology course.
